# Case Report: A case of successful recanalization of chronic total occlusion using a left brachial artery approach for intra-aortic balloon pump support and a right brachial artery–right radial artery approach for bilateral angiography

**DOI:** 10.3389/fcvm.2025.1623034

**Published:** 2025-11-06

**Authors:** Yue Bao, Jun Ma, Hui Guo, Min Wang

**Affiliations:** Department of Cardiology, Wuhan Asia Heart Hospital, Wuhan, China

**Keywords:** iliac artery occlusion, radial artery puncture, brachial artery puncture, chronic total occlusion, intra-aortic balloon pump (IABP)

## Abstract

Chronic total occlusion (CTO) of the coronary arteries can be managed using a retrograde strategy guided by bilateral angiography, substantially improving the likelihood of successful recanalization. To enhance procedural safety, hemodynamic support—such as intra-aortic balloon pump (IABP) counterpulsation—is frequently employed. Nevertheless, in patients presenting with concurrent femoral artery occlusion in the lower limbs, conventional access via the femoral route becomes unfeasible, significantly complicating intervention efforts. This report describes a case involving a patient with CTO alongside complete bilateral external iliac artery occlusion. In this instance, we achieved successful IABP insertion through the left brachial artery and performed bilateral coronary imaging via the right radial and right brachial arteries, ultimately accomplishing effective lesion recanalization. This alternative approach demonstrates a practical solution for individuals with CTO who have limited vascular access in the lower extremities.

## Introduction

The success rate of percutaneous coronary intervention (PCI) for CTO is relatively low, mainly due to the inability of guidewires to pass through the lesion. In recent years, the continuous improvement of traditional devices (such as guidewires, microcatheters, and support catheters), as well as the introduction of new techniques such as the retrograde approach and controlled antegrade and retrograde subintimal tracking (CART), have gradually increased the success rate of CTO-PCI, raising it from 60% to 85%–90% (3, 4). Therefore, it has become routine for patients undergoing PCI for CTO to undergo bilateral angiography to facilitate retrograde access and improve the success rate of guidewire crossing. Among these access options, the radial artery is preferred because of its lower complication rate and shorter recovery time (5). Additionally, PCI for CTO is considered a high-risk procedure, particularly in patients with multivessel coronary artery disease. During the procedure, transient interruption of antegrade flow—caused by contrast injection or balloon dilation—can provoke hemodynamic instability or even cardiac arrest. Consequently, these patients are categorized as having “complex high-risk indicated PCI” (CHIP) CTO. The 2015 SCAI/ACC/HFSA/STS consensus states that mechanical circulatory support (MCS) must be immediately available during CHIP-PCI to preserve systemic and respiratory hemodynamics and to ensure procedural safety and success (6). The IABP remains the most widely used primary MCS device in domestic catheterization laboratories; it promptly unloads the left ventricle and augments coronary and peripheral perfusion by raising diastolic pressure and lowering systolic impedance. The preferred approach for IABP implantation is via the femoral artery (2). However, this patients with coronary heart disease and severe bilateral lower extremity arterial occlusion, this approach may lead to failure of IABP implantation and necessitate the abandonment of revascularization. In this case, the patient had bilateral lower extremity arterial occlusion, making it impossible to implant an IABP through the femoral artery. Additionally, bilateral angiography was required to complete a complex reverse CTO-PCI. For the first time, we implanted an IABP through the left brachial artery and performed bilateral angiography through the right brachial artery and the right radial artery, successfully achieving complex and high-risk CTO revascularization. This provides a new procedural option for critically ill CHIP patients with lower extremity occlusion and has significant clinical significance.

## Case report

**Figure 1:**
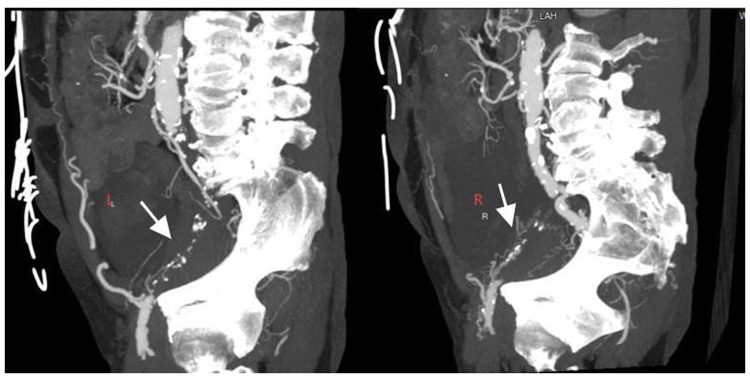
CTA revealing bilateral external iliac artery occlusion (white arrows).

A 74-year-old male was admitted to the hospital due to intermittent chest tightness lasting for 4 years, which had worsened over the past month. The patient has a history of hypertension for more than 10 years. Physical examination of the heart and lungs reveals no abnormalities. On 21 January 2025, coronary angiography performed at an outside hospital revealed 70%–80% stenosis of the left main terminal segment, 60%–70% stenosis in the proximal-to-mid left anterior descending artery (LAD), 99% ostial stenosis of the left circumflex artery (LCX), and complete mid-to-distal occlusion of the right coronary artery (RCA). Echocardiography showed left-ventricular segmental wall-motion abnormalities with an ejection fraction of 40%. Upper-extremity color Doppler ultrasound was normal, whereas lower-extremity computed tomography angiography (CTA) demonstrated bilateral external iliac artery occlusion (Figure 1). Diagnosis: Coronary atherosclerotic heart disease, unstable angina, NYHA functional class II, bilateral external iliac artery occlusion. The patient's coronary artery disease is complex, and the access route for interventional therapy is restricted. Coronary artery bypass grafting is recommended, but the patient refuses. Our treatment plan is to first address the occluded RCA and then schedule the treatment of the non-occluded LCA at a later date. This is because the RCA serves as the recipient vessel, while the LCA acts as the donor vessel providing collateral circulation to the RCA. This approach can reduce the intraoperative risks for the patient.

**Figure 2:**
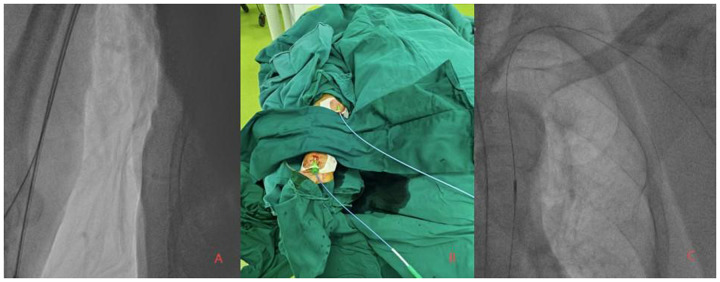
(A,B) bilateral coronary angiography performed via the right radial artery (6Fr sheath) and right brachial artery (7Fr sheath) approaches. **(C)** Fluoroscopic confirmation of successful intra-aortic balloon pump (IABP) placement via the left brachial artery.

**Figure 3:**
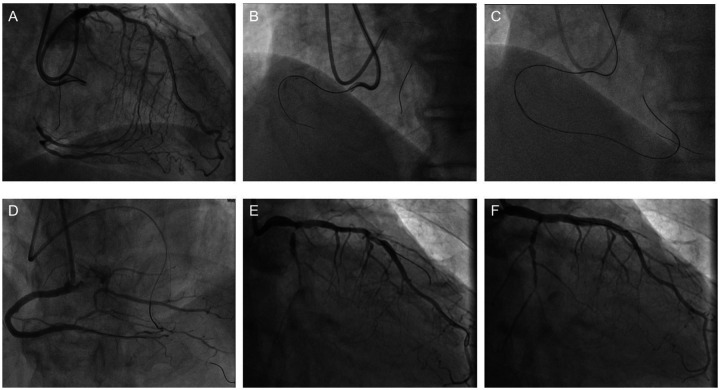
A Bilateral angiography demonstrating total occlusion of the proximal RCA and critical stenosis of the left main coronary artery. **(B)** Failed anterograde guidewire crossing through the RCA occlusion. **(C)** Retrograde recanalization achieved via septal collateral channels. **(D)** Final angiographic result after deployment of four drug-eluting stents in the RCA, with restored TIMI grade 3 flow. **(E)** CAG indicates severe stenosis of the LCA. **(F)** The image of successful stent implantation in the LCA.

**Figure 4:**
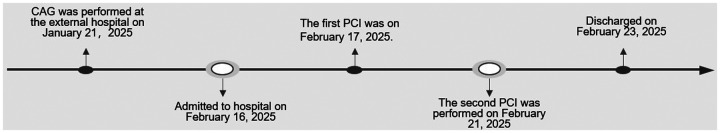
A timeline with relevant data obtained from the episode of care.

The surgical procedure: On February 17, 2025, PCI was performed on the patient with the assistance of IABP. First, punctures were made in the right radial artery and brachial artery, and 6F and 7F arterial sheaths were inserted, respectively (Figures 2A,B). Then, an IABP was placed through the left brachial artery (Figure 2C). A guiding catheter 1 was advanced through the right brachial artery sheath to the ostium of the RCA, and another guiding catheter 2 was advanced through the right radial artery sheath to the ostium of the left main trunk for bilateral angiography (Figure 3A), clearly visualizing the course of the distal RCA. Under the support of microcatheter 1, five guide wires (guide wires 1 and 2: SION Blue AHW14R004S, Asahi; guide wire 3: XT-A APW14R009S, Asahi; guide wires 4 and 5: Gaia First AHW14R007P, Asahi) were sequentially used, but none could cross the occluded segment in the mid-RCA (Figure 3B).Under the support of microcatheter 2, guide wire 2 was advanced through the septal branch–posterior descending (PD) collateral in a retrograde manner into the distal segment of the RCA via guiding catheter 2. Subsequently, guide wires 6, 7, 8, and 9 were sequentially advanced through microcatheter 2 to attempt retrograde recanalization. Guide wire 9 successfully traversed the occluded mid-RCA segment and reached the RCA ostium (Figure 3C). Balloon 2 was used to compress the guide wire, and microcatheter 1 was withdrawn. The extension catheter was advanced into the proximal segment of the RCA through guide catheter 1. Microcatheter 2, along with guide wire 9, was advanced into the extension catheter in the RCA. Guide wire 10 was exchanged in reverse through guide catheter 1. Through guide catheter 1, guide wire 11 was inserted into microcatheter 2. Guide wire 11 was then advanced through microcatheter 2 to the distal segment of the RCA, after which microcatheter 2 was withdrawn. Over guide wire 11, the lesion was pre-dilated sequentially with a balloon, and an Intravascular Ultrasound (IVUS) catheter was advanced to the distal segment of the RCA and pulled back to assess the lesion and guide stent placement. Eventually, four drug-coated stents were successfully implanted (Figure 3D). IVUS assessment showed good stent apposition. Repeat angiography revealed no residual stenosis within the stent and no occlusion of major branches. The procedure proceeded smoothly without any intraoperative complications. On February 21st, the patient underwent left coronary artery intervention again. Two drug-coated stents were implanted in the LM-LAD, and Drug-coated balloon (DCB)dilation was performed in the LCX (Figures 3E,F). The wound healed well after the procedure, and the patient was discharged two days later. After 12 weeks of follow-up, the patient reported no discomfort, and blood circulation in both upper limbs was good. The patient's hospitalization timeline is shown in Figure 4.

## Discussion

Interventional treatment of CTO is often challenging due to the presence of calcified nodules, absence of microchannels, and dense fibrous caps, which make it difficult for guidewires to cross the lesion. Typically, bilateral angiography via the radial artery and a retrograde approach are required to increase the success rate of recanalization (1, 5, 7). Meanwhile, repeated angiography and balloon dilation during the procedure can cause transient no-reflow and a sudden increase in ventricular load, potentially leading to hypotension or malignant arrhythmias. Therefore, circulatory support, such as IABP implantation, is necessary to enhance procedural safety.

This patient had multivessel and CTO with complete occlusion of the bilateral iliac arteries. Traditional femoral artery access was not feasible for IABP placement. We instead implanted the IABP via the left brachial artery and performed bilateral angiography through the right brachial and radial arteries for reverse intervention to open the occluded lesions. Stents were successfully implanted, and IVUS confirmed complete stent apposition (8). TIMI grade 3 flow was achieved. After the removal of the upper limb artery catheter postoperatively, manual compression combined with pressure bandaging was used for hemostasis. No hematoma or arterial occlusion occurred at the puncture site. During the 12-week postoperative follow-up, the arterial pulsation of both upper limbs was good, and there were no puncture-related hematomas or neurological complications. This study was a single-center case without a control group or long-term follow-up. The risks of brachial artery IABP-related thrombosis, upper limb ischemia, or nerve injury still need to be further verified by large-sample, multi-center studies. In the future, for complex CTO-PCI patients with lower extremity arterial occlusion, the upper limb approach can be considered as an alternative option for interventional treatment.

## Data Availability

The raw data supporting the conclusions of this article will be made available by the authors, without undue reservation.
